# Pulse pressure mediation and hypertension modulation: the impact of TyG index on renal function deterioration in CKD patients under the CKM syndrome framework

**DOI:** 10.3389/fendo.2026.1809079

**Published:** 2026-04-17

**Authors:** Xianqiong Zhu, Xinze Liu, Jiaqi An, Qiaoya He, Sheng Wang, Zheng Zhang, Yue Yang, Li Zhuo, Wenge Li

**Affiliations:** 1 Department of Nephrology, China-Japan Friendship Hospital, Beijing, China; 2 Peking University China-Japan Friendship School of Clinical Medicine, Beijing, China; 3 China-Japan Friendship Clinic Medical College, Beijing University of Chinese Medicine, Beijing, China; 4 Institute of Clinical Medical Sciences, China-Japan Friendship Hospital, Beijing, China

**Keywords:** cardiovascular-kidney-metabolic (CKM) syndrome, chronic kidney disease (CKD), pulse pressure (PP), renal function deterioration, triglyceride-glucose (TyG) index

## Abstract

**Background:**

Under the Cardiovascular-Kidney-Metabolic (CKM) syndrome framework, insulin resistance (IR) is the core driver of renal damage. The triglyceride-glucose (TyG) index is a validated IR surrogate, but existing evidence is mostly limited to cross-sectional data or single-source databases, failing to clarify the longitudinal progression of renal injury and underlying mechanisms.

**Methods:**

This study integrated two independent prospective cohorts: the national community-based CHARLS cohort (4,476 middle-aged and elderly participants, 2011–2015 follow-up) and a hospital-based KDIGO 2012-defined CKD cohort (396 patients, median 1,019-day follow-up). We analyzed the association of TyG index with eGFR decline (CHARLS) and ESRD (CJFH), with pulse pressure as mediator, hypertension as moderator, via regression, mediation, survival and nonlinear analyses.

**Results:**

Elevated baseline TyG index was independently associated with renal function deterioration in both cohorts. Pulse pressure played a partial mediating role in this association, while baseline hypertension significantly amplified the adverse renal effect of TyG. The overall association was dominated by a negative linear trend with no significant risk threshold.

**Conclusion:**

TyG index is an independent risk factor for renal function deterioration, with its adverse effects partially mediated by elevated pulse pressure and amplified by hypertension. This study provides epidemiological evidence for the CKM “metabolism-vascular-renal” axis, supporting TyG-based early renal risk stratification in the general population and multi-dimensional interventions for CKD patients.

## 1 Background

Chronic kidney disease (CKD) has emerged as a paramount global public health challenge, characterized by a burgeoning prevalence and an intimate association with heightened risks of cardiovascular events and all-cause mortality. Traditionally, clinical management paradigms for CKD have concentrated on the rigorous control of established independent risk factors, primarily diabetes mellitus and hypertension (1). However, a paradigm shift is occurring as accumulating evidence suggests that diabetes, cardiovascular disease (CVD), and CKD are not merely comorbid conditions but are deeply intertwined pathophysiologically, forming a singular, complex syndrome. In response, the American Heart Association (AHA) has proposed the cardiovascular-kidney-metabolic (CKM) syndrome framework. This integrative framework seeks to encompass the continuous pathological trajectory from early metabolic risk factors to multi-organ dysfunction and end-stage damage.

Within the CKM syndrome framework, insulin resistance (IR) is recognized as a core pathophysiological hub that drives the development and progression of the entire spectrum of metabolic and vascular diseases. IR exerts deleterious effects on both the cardiovascular and renal systems through a multitude of pathways, including the induction of systemic chronic inflammation, enhanced oxidative stress, and the promotion of endothelial dysfunction. The triglyceride-glucose (TyG) index, a composite marker derived from fasting triglycerides and blood glucose levels, has been widely validated as a simple, reliable, and cost-effective surrogate for insulin resistance. Multiple cross-sectional studies have demonstrated a robust association between the TyG index and the prevalence of CKD (2). However, the cross-sectional nature of these prior investigations limits their capacity to establish a clear temporal sequence or define the causal directionality between insulin resistance and the decline in renal function (3). Moreover, most studies have relied on single-point eGFR measurements, which may not capture the long-term trajectory toward “Renal Function Deterioration”—a concept that necessitates the unification of functional decline (eGFR slope) and terminal structural failure (End-Stage Renal Disease, ESRD).

According to the theoretical foundations of CKM syndrome, the damage inflicted by metabolic abnormalities on the renal parenchyma is rarely a direct singular event. Instead, it often propagates through a cascade of cardiovascular injuries, manifesting as the “metabolism-vascular-renal” axis. Pulse pressure (PP), defined as the difference between systolic and diastolic blood pressure, serves as a clinical reflection of aortic stiffness and increased peripheral vascular resistance (4). Elevated pulse pressure is a potent predictor of cardiovascular risk and has been hypothesized to act as a “mechanical transmission medium” that translates systemic metabolic insults into intrarenal hemodynamic stress. Theoretically, insulin resistance exacerbates arterial wall remodeling and stiffening, leading to widened pulse pressure, which then increases the pulsatile load on the renal microcirculation. Nevertheless, empirical evidence supporting this specific longitudinal pathway—linking the TyG index to renal outcomes through pulse pressure—remains insufficient, representing a key research gap.

Furthermore, hypertension is perhaps the most ubiquitous comorbidity within the CKM syndrome. It is well-established as a risk factor for CKD progression, but its potential role as a moderator—specifically how it might interact with or amplify the nephrotoxic effects of insulin resistance—is still poorly understood. Population-level evidence exploring this moderating effect is currently insufficient to guide personalized clinical interventions. To address these critical scientific questions, this study adopts a dual-evidence strategy. We first utilize data from the China Health and Retirement Longitudinal Study (CHARLS), a large-scale national prospective cohort of community-dwelling middle-aged and elderly population, to explore the predictive value of TyG index for early renal function decline in the general population. We then validate the causal association and pathophysiological mechanism using an independent clinical cohort of CKD patients strictly in accordance with the KDIGO 2012 CKD diagnostic criteria from the China-Japan Friendship Hospital (CJFH).

The rationale for this combined approach is rooted in the need for both statistical power and clinical precision. Large public databases like CHARLS provide a vast, objective sample size of general middle-aged and elderly population, which allows for the rigorous control of confounding variables and the identification of the predictive value of TyG index for early renal function decline in the pre-disease state, while ensuring sufficient sample size for statistical analysis. However, public databases can be susceptible to heterogeneity in measurement and the absence of hard clinical endpoints. To resolve this, the independent clinical cohort at CJFH provides a real-world validation in high-risk CKD patients confirmed by KDIGO criteria, focusing on the definitive outcome of ESRD (dialysis or kidney transplantation). By unifying the dependent variables as “Renal Function Deterioration,” we aim to provide a more comprehensive and clinically actionable understanding of the CKM axis. This study adopts a two-stage exploratory-validation design with two independent cohorts: we first utilize the national prospective CHARLS cohort to explore the predictive value of TyG index for early renal function decline in community-dwelling middle-aged and elderly general population; we then validate the association and pathophysiological pathways in an independent clinical cohort of KDIGO 2012-defined CKD patients from CJFH. This is the first study to longitudinally analyze the association between TyG index and renal function deterioration across the full spectrum from general population to confirmed CKD patients in China, while exploring the mediating role of pulse pressure and moderating effect of hypertension in both settings (5). Our ultimate goal is to offer stratified insights and epidemiological evidence for early renal impairment risk stratification in general population and multi-dimensional intervention strategies for CKD patients at high risk of kidney failure, based on the inherent heterogeneity of different populations.

## 2 Methods

### 2.1 Study population and sample selection

This research adopts an exploratory-validation two-stage design with two independent datasets, to clarify the association between TyG index and renal function deterioration across different populations and disease stages, while fully considering the inherent heterogeneity between cohorts. The first is the CHARLS cohort, a large national prospective community-based cohort used for exploratory analysis of TyG index in predicting early renal function decline in middle-aged and elderly general population; the second is the CJFH cohort, a single-center hospital-based cohort of KDIGO 2012-defined CKD patients, used for clinical validation of TyG index in predicting progression to hard renal endpoint, with clear differences in population characteristics, follow-up design, outcome indicators and covariate adjustment strategies between the two cohorts.

#### 2.1.1 Public discovery cohort: CHARLS

The discovery phase of this study utilized data from the China Health and Retirement Longitudinal Study (CHARLS), a nationally representative prospective cohort of community-dwelling individuals aged ≥ 45 years. We utilized data from the 2011 baseline, 2013 mid-term follow-up, and 2015 endpoint follow-up. The primary purpose of this cohort was to explore the predictive value of baseline TyG index for long-term renal function decline in the general middle-aged and elderly population, rather than focusing on confirmed CKD patients.

Inclusion criteria (1): Availability of fasting triglyceride and fasting blood glucose test data in 2011 (for calculating the TyG index) (2); Complete eGFR test data in both 2011 and 2015 (3); Follow-up duration ≥4 years with no missing key variables.

Exclusion criteria: (1) Diagnosis of end-stage renal disease (ESRD) at baseline in 2011, including eGFR <15 mL/min/1.73 m², receipt of kidney transplantation, or dialysis treatment; (2) Estimated glomerular filtration rate (eGFR) >120 mL/min/1.73 m²; (3) Death from non-CKD-related factors such as malignant tumors and acute cardio-cerebrovascular events between 2011 and 2015; (4) Missing key variables (e.g., TyG index, pulse pressure, hypertension status); (5) Obvious logical errors or outliers in the data (e.g., TyG index <5 or >15). A total of 4,476 participants were included in the CHARLS analysis.

Limitations note: Urinary albumin data were not fully available in the CHARLS database, and CKD status was not confirmed by repeated eGFR measurements or kidney injury markers as required by the KDIGO guidelines. Meanwhile, the CHARLS database does not provide complete data on whether participants took antihypertensive drugs, statins, ezetimibe, and whether they had hypothyroidism, so the above factors could not be included in the screening and adjustment for this cohort. We have adjusted for drinking status as a covariate in all statistical models of this cohort to control the potential confounding effect of alcohol intake. Therefore, the included participants were community-dwelling general population without confirmed CKD diagnosis, which is essentially different from the CJFH cohort of KDIGO-defined CKD patients. The results of this cohort were only used for exploratory analysis of early renal function decline prediction in the general population, and cannot be directly extrapolated to confirmed CKD patients.

#### 2.1.2 Clinical validation cohort: CJFH

To validate the findings in a high-risk clinical context, we utilized an independent cohort of CKD patients strictly in accordance with the KDIGO 2012 CKD diagnostic criteria from the China-Japan Friendship Hospital. This approach addresses the common criticism of database-only research by providing “real-world” clinical verification in patients at highest risk for terminal renal failure.

Inclusion criteria: (1) Age ≥ 45 years; (2) confirmed diagnosis of CKD in line with KDIGO 2012 guidelines, with baseline eGFR <60 mL/min/1.73 m² confirmed by repeated measurements for ≥3 months; (3) complete baseline data for TyG index, pulse pressure, hypertension status, and urinary albumin-related indicators; (4) documented ESRD outcomes (event status and time to event).

Exclusion criteria: (1) Baseline ESRD; (2) baseline eGFR ≥60 mL/min/1.73 m²; (3) taking antihypertensive drugs, statins, or ezetimibe at baseline; (4) diagnosed with hypothyroidism; (5) missing crucial variables; (6) follow-up < 3 months. A total of 396 patients were included, with a median follow-up of 1,019 days (January 2014 to December 2021). All core laboratory indicators (including TyG index-related fasting triglyceride and blood glucose, pulse pressure, and covariates) were only detected at baseline admission, and no repeated laboratory testing was performed during follow-up. The follow-up only focused on the confirmation of the hard endpoint (ESRD occurrence and occurrence time), which is a classic design for prospective cohort studies with clinical endpoint events.

### 2.2 Variable definitions

#### 2.2.1 Core exposure: TyG index

TyG index: Calculated using the classic formula: TyG index = ln [fasting triglyceride (TG, mg/dL) × fasting blood glucose (FBG, mg/dL)/2]; Fasting TG and FBG were obtained from baseline fasting venous blood tests in 2011. In the CJFH cohort, TyG was analyzed both as a continuous variable and categorized into tertiles (T1: < 8.44; T2: 8.45–9.12; T3: > 9.12) to assess dose-response effects.

#### 2.2.2 Outcome: Renal Function Deterioration

##### 2.2.2.1 Functional Outcome (ΔeGFR)

In the CHARLS cohort, eGFR was calculated using the CKD-EPI (Cr-CysC) combined formula (6). Deterioration was measured as the magnitude of change: ΔeGFR = eGFR in 2015 - eGFR in 2011.

##### 2.2.2.2 Structural/Hard Outcome (ESRD)

In the CJFH clinical cohort, deterioration was defined as the occurrence of End-Stage Renal Disease, necessitating permanent dialysis or kidney transplantation.

#### 2.2.3 Mediator and Moderator

##### 2.2.3.1 Pulse Pressure

Defined as Systolic Blood Pressure (SBP) - Diastolic Blood Pressure (DBP). In CHARLS, PP at 2013 was used as a mid-term mediator. In CJFH, baseline PP was utilized to adjust the hazard models for ESRD.

##### 2.2.3.2 Hypertension

Baseline status defined by SBP ≥ 140 mmHg or DBP ≥ 90 mmHg, self-reported physician diagnosis.

#### 2.2.3 Covariates

Demographic factors: Gender, age, place of residence (rural/urban), educational level (illiterate/primary school/junior high school/senior high school and above), marital status (with spouse/without spouse); Lifestyle factors: BMI (weight/height², kg/m²), smoking status (current smoker/never smoked/former smoker), drinking status (current drinker/never drank/former drinker); Disease history factors: Diabetes mellitus (self-reported diagnosis, fasting blood glucose ≥7.0 mmol/L), hyperlipidemia (self-reported diagnosis, TG ≥2.26 mmol/L, or LDL-C ≥4.14 mmol/L), stroke, heart disease, liver disease, malignant tumor, chronic disease history; Medical-related factors: History of hospitalization between 2011 and 2015, frequency of outpatient visits, and whether medical insurance was purchased. For the CJFH clinical cohort, variables were further classified by causal pathway: ① Pre-exposure confounders: demographic factors, smoking/drinking status, disease history (except diabetes), medical-related factors; ② Potential parallel mediators: BMI, diabetes mellitus, continuous HDL and LDL concentrations (replacing categorical dyslipidemia to reduce information loss).

### 2.3 Statistical Analysis

All analyses were conducted using R 4.3.1. Statistical significance was defined as a two-tailed P < 0.05.

#### 2.3.1 Community-based discovery cohort (CHARLS)

##### 2.3.1.1 Association analysis

Stratified trend analysis (TyG index quartile grouping), continuous association analysis (scatter plot and linear fitting trend line), and multiple linear regression (stepwise inclusion of covariates) were used to analyze the predictive effect of the TyG index on ΔeGFR.

##### 2.3.1.2) Sensitivity analysis

This part is presented in the Appendix. The reason for conducting comprehensive sensitivity analysis only in the Community-based Discovery Cohort is that the CHARLS cohort is a community-based population with higher heterogeneity in demographic characteristics, lifestyle factors, and medical resource access. Therefore, multiple sensitivity analyses (replacement of dependent variable measurement methods, inclusion of urban fixed effects, restriction of sample range, double machine learning model) were required to verify the robustness of the results. In contrast, the Hospital-based Clinical Validation Cohort consists of hospitalized CKD patients with more homogeneous clinical conditions and higher data quality, so additional sensitivity analysis was not performed.

##### 2.3.1.3 Mediating effect analysis

Two complementary and standardized causal mediation analysis methods were used to explore the potential mediating role of pulse pressure, with all models adjusted for baseline eGFR (2011), baseline pulse pressure (2011) and the full set of pre-specified covariates:①Time-anchored structural equation model (SEM): Based on the time sequence of “2011 TyG index → 2013 pulse pressure → 2011-2015 ΔeGFR”, a time-anchored SEM was constructed to estimate the path coefficients, indirect effect, total effect and the proportion mediated. Standard errors, 95% confidence intervals and significance tests were derived from 1000 bootstrap replications.②Counterfactual framework-based mediation analysis: Under the counterfactual causal inference framework, the controlled direct effect (CDE), natural direct effect (NDE), natural indirect effect (NIE) and marginal total effect (MTE) were estimated. Bias-corrected 95% confidence intervals were obtained via 1000 bootstrap replications to correct finite-sample sampling bias and improve the robustness of the results.

##### 2.3.1.4 Moderating effect analysis

The sample was divided into hypertension and non-hypertension groups. Multiple linear regression models were constructed for each group, and the Bootstrap method (1000 repeated samplings) was used to test the statistical significance of coefficient differences.

##### 2.3.1.5 Nonlinear analysis

A multiple linear regression model incorporating the quadratic term of the TyG index was constructed to analyze the nonlinear dose-response relationship.

#### 2.3.2 Hospital-based clinical validation cohort (CJFH)

##### 2.3.2.1 Survival analysis

Kaplan-Meier (KM) method was used to draw the cumulative risk curve of ESRD, and the Log-rank test was used to compare differences between groups.

##### 2.3.2.2 Cox proportional hazards regression

Hierarchical Cox models were constructed to estimate two predefined causal parameters:① Model 1 (crude model): demographic variables; ② Model 2 (Total effect model): adjusted only for pre-exposure confounders, to estimate the complete total effect of TyG index on ESRD without blocking potential mediated pathways; ③ Model 3 (Direct effect model): further adjusted for potential parallel mediators (BMI, diabetes, HDL, LDL) on the basis of Model 2, to estimate the direct effect independent of competing pathways, which served as the baseline model for subsequent pulse pressure mediation analysis.

##### 2.3.2.3 Mediating effect verification

To isolate the independent mediating effect of pulse pressure and eliminate interference from competing mediated pathways, verification was performed based on Model 3 (direct effect model). Pulse pressure was added to Model 3 to construct Model 4, and changes in the TyG index’s HR were compared.

##### 2.3.2.4 Moderating effect verification

Cox regression models were constructed by stratifying according to hypertension status, and differences in the HR of the TyG index between the two groups were compared.

##### 2.3.2.5 Nonlinear test

Restricted cubic spline (RCS) terms of the TyG index were included to test the nonlinear relationship.

## 3 Results

### 3.1 Causal inference framework

**Figure 1:**
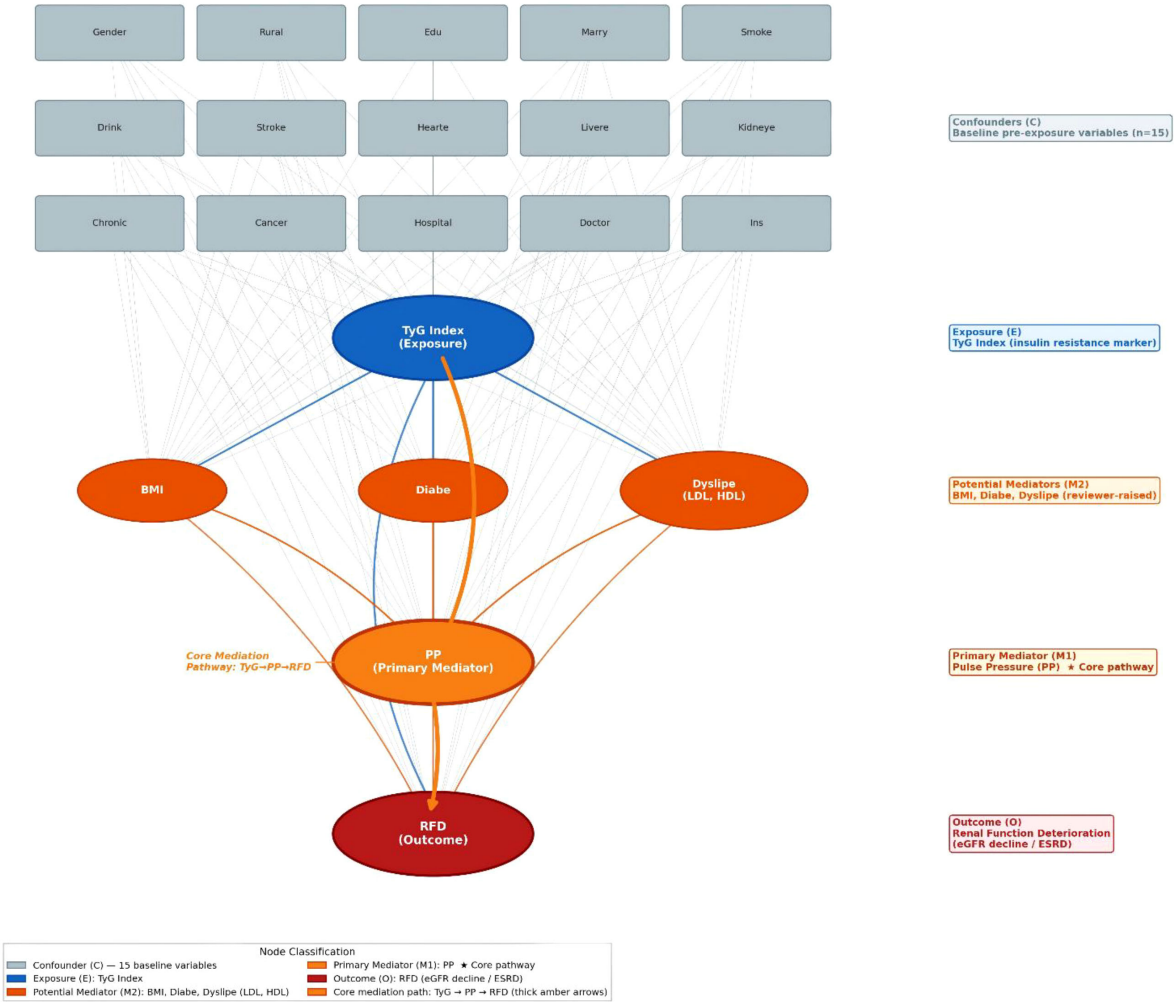
Directed Acyclic Graph (DAG) The total effect of TyG on RFD is estimated by adjusting only for pre-specified baseline confounders, which closes all backdoor paths while fully retaining all potential mediated pathways to capture the complete causal effect. For mechanism analysis of the core hypothesized pathway (TyG → pulse pressure [PP] → RFD), the controlled direct effect is estimated by additional adjustment for parallel mediators (body mass index, diabetes mellitus, low-density lipoprotein cholesterol [LDL], high-density lipoprotein cholesterol [HDL]). This adjustment blocks competing indirect pathways from these independent parallel mediators, eliminates interference from overlapping mediated effects, and thus isolates and accurately quantifies the specific, independent mediating effect of PP. As a validated surrogate of insulin resistance, elevated TyG index increases PP by promoting arterial stiffness and endothelial dysfunction; elevated PP further impairs renal autoregulation, accelerates glomerular injury, and ultimately drives estimated glomerular filtration rate decline and end-stage renal disease progression. The proportion of RFD risk mediated by PP is calculated via formal causal mediation analysis adjusted for all baseline confounders and the above parallel mediators.

To explicitly define the causal inference framework of this study and standardize the analytical logic for subsequent effect estimation, we constructed a directed acyclic graph (DAG) based on the pathophysiological core of the cardiovascular-kidney-metabolic syndrome framework (**Figure 1**). This DAG clarifies our preset causal assumptions, variable classification rules, and covariate adjustment strategies for different effect targets, providing a rigorous methodological basis for all subsequent statistical analyses.

### 3.2 Association between TyG index and renal function deterioration

#### 3.2.1 Community-based Discovery Cohort (CHARLS)

**Figure 2:**
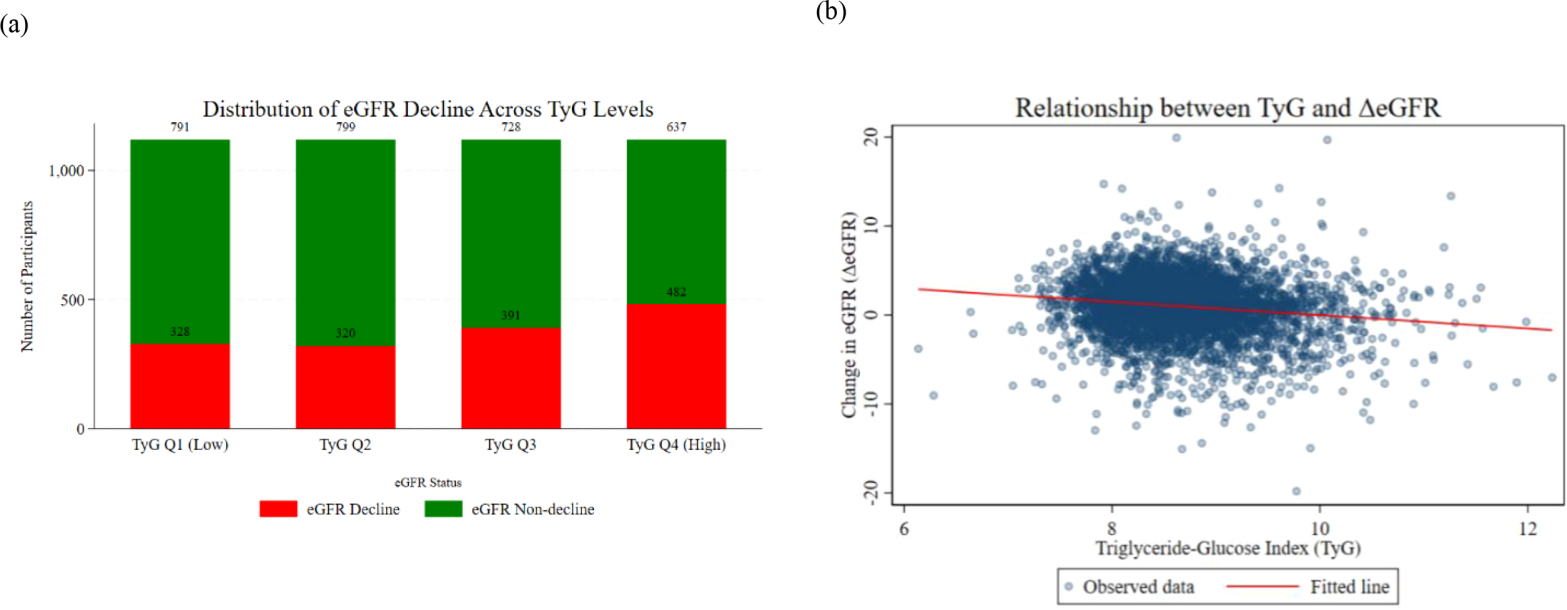
Distribution and association of TyG index with eGFR changes in the CHARLS cohort **(a)** Number distribution of participants with eGFR decline (red) and non-decline (green) after quartile stratification of the 2011 TyG index (Q1: low, Q2, Q3, Q4: high) in the CHARLS cohort. The proportions of eGFR decline in Q1 to Q4 were 29.3%, 28.5%, 34.9%, and 43.1%, respectively, showing a stepwise increasing trend with higher TyG quartiles. **(b)** Scatter plot and linear fitted line illustrating the relationship between the TyG index and ΔeGFR (2011–2015) in the CHARLS cohort. Observed data points are distributed around the fitted line without obvious abnormal deviations, indicating a linear association trend.

**Figure 3:**
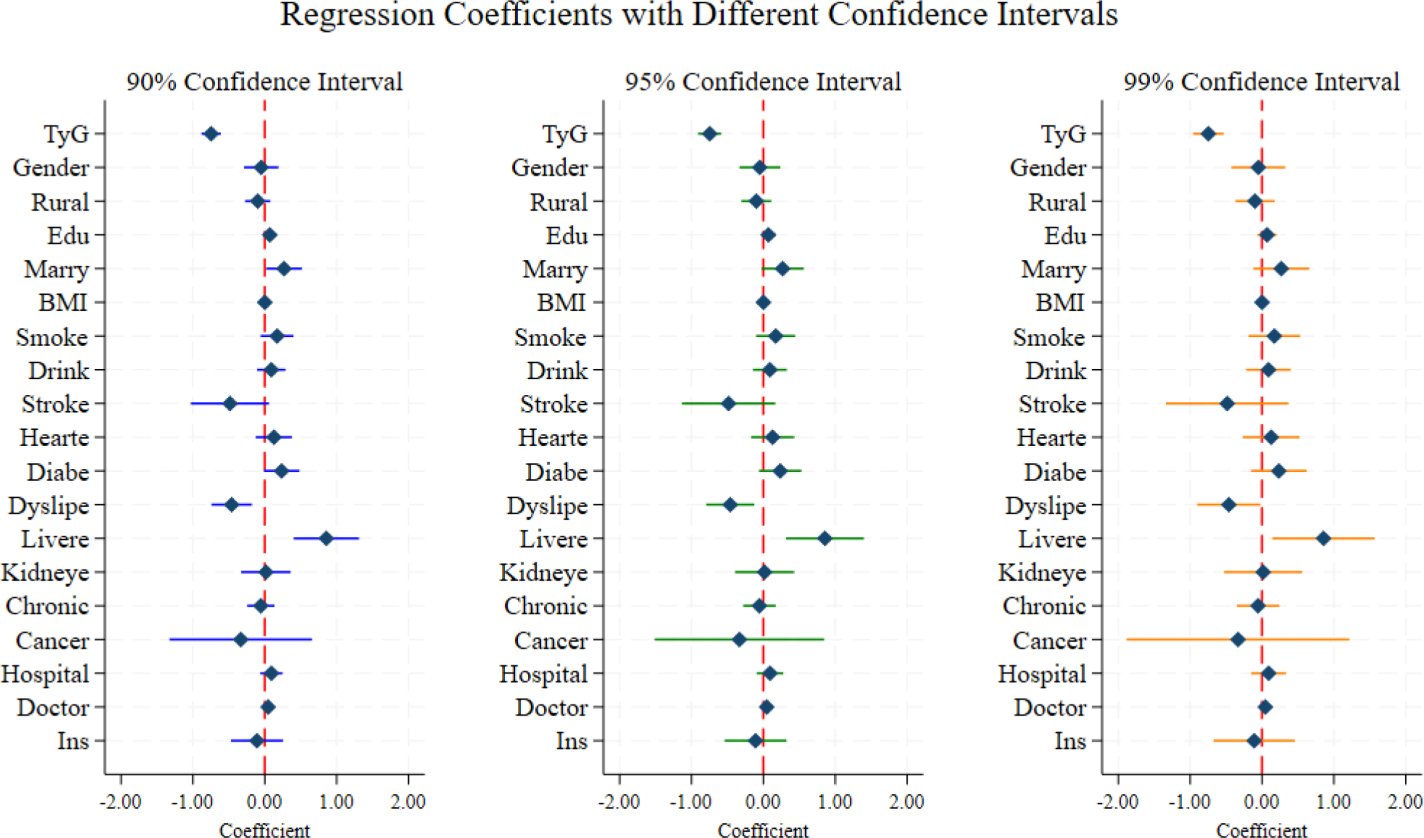
Distribution of regression coefficients and confidence intervals in the multiple linear regression model (CHARLS cohort) This figure presents the regression coefficients and their 90%, 95%, and 99% confidence intervals for each variable in the multiple linear regression model, with ΔeGFR as the dependent variable and the 2011 TyG index as the core independent variable. Covariates included demographic factors, lifestyle factors, disease history, and medical-related factors. The key result is that the confidence intervals of the TyG index did not include 0 at all three confidence levels, with a negative regression coefficient (β=-0.736) and statistical significance (P<0.001). An increase in the TyG index was significantly associated with a decrease in ΔeGFR, and this association remained stable across the three confidence levels.

In the quartile grouping of the 2011 TyG index, the proportions of community-dwelling middle-aged and elderly participants with eGFR decline in Q1 to Q4 groups were 29.3%, 28.5%, 34.9%, and 43.1% respectively, showing a stepwise increase (**Figure 2a**). Continuous association analysis showed that the scatter points were distributed around the fitted line, with no obvious abnormal deviation (**Figure 2b**). Multiple linear regression results showed that after adjusting for all covariates, the TyG index was significantly negatively associated with ΔeGFR under 90%, 95%, and 99% confidence levels (P<0.05), indicating that a higher TyG index was associated with a greater magnitude of eGFR decline (**Figure 3**).

#### 3.2.2 Hospital-based Clinical Validation Cohort (CJFH)

**Figure 4:**
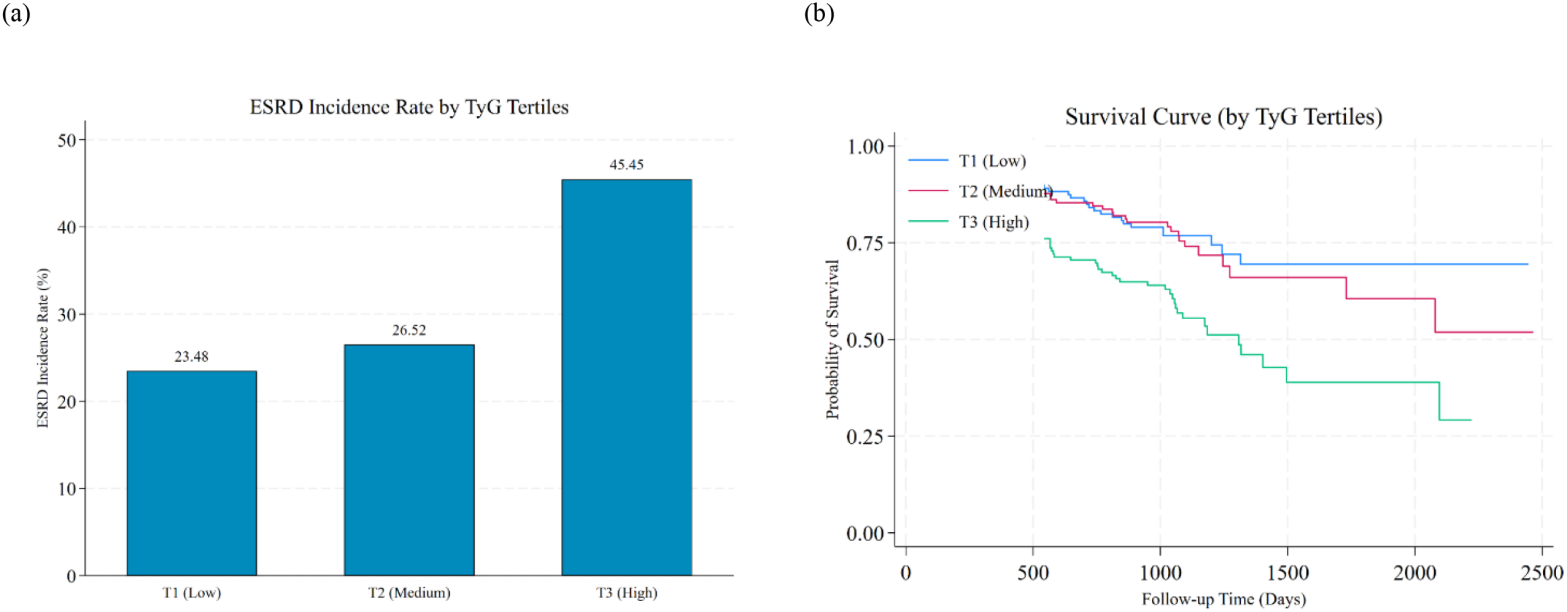
Distribution and association of TyG index with ESRD risk in the CJFH cohort **(a)** ESRD incidence rates across TyG tertiles (T1: low, T2: medium, T3: high) in the CJFH cohort. The incidence rates were 23.48% (T1), 26.52% (T2), and 45.45% (T3), respectively, showing a clear stepwise upward trend with increasing TyG levels. **(b)** Kaplan-Meier survival curves for ESRD-free survival stratified by TyG tertiles in the CJFH cohort. The survival probability of the high TyG group (T3) declined most steeply, and the Log-rank test confirmed significant differences in survival curves between groups (P = 0.0001).

The ESRD incidence rates in the TyG tertile groups (T1: low, T2: medium, T3: high) among KDIGO-defined CKD patients were 23.48%, 26.52%, and 45.45% respectively, showing a clear stepwise trend (**Figure 4a**). Kaplan-Meier survival curves showed that the survival probability of the high TyG group (T3) decreased most steeply, and the Log-rank test confirmed significant differences between groups (P = 0.0001) (**Figure 4b**). Hierarchical Cox regression showed consistent associations between TyG index and ESRD risk: ① Total effect (Model 2): each 1-unit TyG increase was associated with a 66.2% higher ESRD risk (HR = 1.662, 95% CI [1.293,2.138], P<0.001), representing the complete effect of TyG without dilution; ② Direct effect (Model 3): after adjusting for potential parallel mediators, each 1-unit TyG increase was still associated with a 60.6% higher ESRD risk (HR = 1.606, 95% CI [1.196,2.155], P<0.01) (**Table 1**). For categorical analysis, compared with T1, the HR of ESRD in T3 was 1.843 (95% CI [1.143,2.972], P<0.05) in Model 3, while T2 showed no statistical significance (P>0.05) (**Table 2**).

**Table 1:** Cox Regression: TyG Index (Continuous) and ESRD Risk in the CJFH Cohort.

Variable	(1)Model 1		(2)Model 2		(3)Model 3	
	Hazard Ratio	95% confidence intervals	Hazard Ratio	95% confidence intervals	Hazard Ratio	95% confidence intervals
TyG	1.688	[1.333,2.138]	1.662	[1.293,2.138]	1.606	[1.196,2.155]
gender	1.536	[1.002,2.356]	1.570	[1.012,2.435]	1.204	[0.762,1.902]
Rural	0.961	[0.642,1.438]	1.041	[0.685,1.583]	0.835	[0.539,1.294]
Edu	0.797	[0.658,0.966]	0.811	[0.664,0.991]	0.824	[0.668,1.016]
Marry	1.051	[0.610,1.808]	0.927	[0.530,1.622]	0.870	[0.492,1.540]
Smoke			0.893	[0.583,1.365]	0.924	[0.590,1.448]
Drink			1.213	[0.797,1.847]	1.075	[0.697,1.656]
Stroke			1.916	[0.863,4.254]	1.728	[0.761,3.926]
Hearte			0.743	[0.417,1.324]	0.793	[0.446,1.410]
Livere			0.493	[0.170,1.430]	0.440	[0.153,1.266]
Kidneye			1.689	[0.867,3.292]	1.523	[0.788,2.941]
Chronic			0.967	[0.631,1.481]	1.031	[0.666,1.595]
Cancer			1.768	[0.702,4.456]	1.623	[0.641,4.111]
Hospital			0.944	[0.579,1.540]	0.824	[0.492,1.378]
Doctor			1.079	[0.972,1.197]	1.054	[0.943,1.178]
Ins			0.845	[0.338,2.112]	0.951	[0.376,2.404]
BMI					1.092	[1.058,1.127]
Diabe					1.067	[0.642,1.771]
HDL					0.356	[0.200,0.635]
LDL					1.161	[1.020,1.321]
*N*	396		396		396	

Model 1: Crude model, Adjusted for demographic variables (gender, rural residence, education level, marital status); Model 2 (Total effect model): Adjusted only for pre-exposure confounders, to estimate the complete total effect of TyG index on ESRD; Model 3 (Direct effect model): Further adjusted for potential parallel mediators (BMI, diabetes, HDL, LDL) on the basis of Model 2, to estimate the independent direct effect and serve as the baseline model for mediation analysis.

**Table 2:** Cox Regression: TyG Index (Tertiles) and ESRD Risk in the CJFH Cohort.

Variable	(1)Model 1		(2)Model 2		(3)Model 3	
	Hazard Ratio	95% confidence intervals	Hazard Ratio	95% confidence intervals	Hazard Ratio	95% confidence intervals
1.TyG_group	1	[1,1]	1	[1,1]	1	[1,1]
2.TyG_group	1.098	[0.674,1.789]	1.112	[0.679,1.822]	1.076	[0.655,1.767]
3.TyG_group	2.246	[1.443,3.495]	2.225	[1.404,3.528]	1.843	[1.143,2.972]
gender	1.593	[1.042,2.437]	1.625	[1.048,2.520]	1.244	[0.788,1.965]
Rural	0.985	[0.658,1.475]	1.078	[0.709,1.640]	0.849	[0.547,1.317]
Edu	0.780	[0.641,0.949]	0.789	[0.643,0.968]	0.818	[0.661,1.011]
Marry	1.061	[0.616,1.827]	0.942	[0.536,1.653]	0.892	[0.504,1.579]
Smoke			0.911	[0.594,1.398]	0.928	[0.590,1.460]
Drink			1.197	[0.786,1.823]	1.103	[0.716,1.700]
Stroke			2.013	[0.907,4.468]	1.805	[0.795,4.100]
Hearte			0.814	[0.460,1.440]	0.815	[0.457,1.455]
Livere			0.471	[0.163,1.359]	0.438	[0.152,1.262]
Kidneye			1.759	[0.914,3.388]	1.465	[0.756,2.841]
Chronic			0.917	[0.598,1.407]	0.954	[0.617,1.474]
Cancer			1.654	[0.655,4.178]	1.504	[0.596,3.793]
Hospital			0.934	[0.578,1.509]	0.805	[0.487,1.331]
Doctor			1.093	[0.982,1.216]	1.057	[0.944,1.183]
Ins			0.826	[0.331,2.064]	0.909	[0.359,2.300]
BMI					1.091	[1.057,1.126]
Diabe					1.276	[0.799,2.038]
HDL					0.351	[0.196,0.629]
LDL					1.184	[1.044,1.343]
*N*	396		396		396	

Reference group: TyG T1 (low, <8.44). Model 1: Crude model, Adjusted for demographic variables (gender, rural residence, education level, marital status); Model 2 (Total effect model): Adjusted only for pre-exposure confounders, to estimate the complete total effect of TyG index on ESRD; Model 3 (Direct effect model): Further adjusted for potential parallel mediators (BMI, diabetes, HDL, LDL) on the basis of Model 2, to estimate the independent direct effect and serve as the baseline model for mediation analysis.

### 3.3 Mediating effect of pulse pressure

#### 3.3.1 Community-based Discovery Cohort (CHARLS)

We adopted two standardized causal mediation analysis methods to explore the potential mediating role of pulse pressure in the association between TyG index and ΔeGFR, with all models adjusted for baseline eGFR (2011), baseline pulse pressure (2011) and the full set of pre-specified covariates.

The time-anchored structural equation model (SEM) based on the preset time sequence showed excellent model fit (CFI = 1.000, TLI = 1.000, RMSEA = 0.000, SRMR = 0.000). Path analysis results showed that the 2011 TyG index significantly positively predicted 2013 pulse pressure (β=1.105, 95%CI [0.156, 2.054], P = 0.022), and 2013 pulse pressure significantly inversely predicted ΔeGFR (β=-0.011, 95%CI [-0.018, -0.004], P = 0.002). The total effect of TyG index on ΔeGFR was β=-0.691 (P<0.001), the indirect effect via pulse pressure was β=-0.012 (95%CI [-0.025, 0.000], P = 0.053), and the proportion mediated was 1.77% (**Table 3**).

**Table 3:** Path coefficients and mediation effect estimates from the time-anchored structural equation model.

Path/Effect	Estimate	Std Err	P>|z|	95% Conf Interval Lower	95% Conf Interval Upper
TyG_2011→PP_2013	1.105251	0.4842101	0.022	0.1562166	2.054285
PP_2013→ΔeGFR	-0.011063	0.003585	0.002	-0.0180894	-0.0040366
TyG_2011→ΔeGFR	-0.6787095	0.0999672	0.000	-0.8746417	-0.4827773
Indirect Effect	-0.0122274	0.0063299	0.053	-0.0246337	0.0001789
Total Effect	-0.6909369	0.1000843	0.000	-.8870986	-.4947752
Proportion Mediated	0.0176968	0.0093886	0.059	-0.0007046	0.0360981

This table reports the path coefficients, indirect effect, total effect, and proportion mediated from a time-anchored structural equation model (SEM) examining the mediating role of 2013 pulse pressure (PP_2013) in the association between the triglyceride-glucose index (TyG_2011) and the change in estimated glomerular filtration rate (ΔeGFR). The model adjusts for baseline 2011 pulse pressure (PP_2011), baseline 2011 estimated glomerular filtration rate (eGFR_2011), and pre-specified covariates. Maximum likelihood estimation was applied, with standard errors, 95% confidence intervals (CIs), and significance tests derived from 1000 bootstrap replications. The model exhibited excellent overall fit, with core representative fit statistics as follows: Comparative Fit Index (CFI) = 1.000, Tucker-Lewis Index (TLI) = 1.000, root mean squared error of approximation (RMSEA) = 0.000 (90% CI: 0.000, 0.000; pclose = 1.000), standardized root mean squared residual (SRMR) = 0.000, Akaike’s Information Criterion (AIC) = 186344.075, and Bayesian Information Criterion (BIC) = 186635.836.

Further counterfactual framework-based mediation analysis confirmed the robustness of the above results. After bias correction via 1000 bootstrap replications, the natural indirect effect (NIE) of TyG index on ΔeGFR through pulse pressure was β=-0.022 (95%CI [-0.056, -0.004], P = 0.043), which was statistically significant. The natural direct effect (NDE) was β=-0.881 (95%CI [-1.164, -0.627], P<0.001), and the marginal total effect (MTE) was β=-0.902 (95%CI [-1.181, -0.643], P<0.001) (**Table 4**). The above results consistently indicated that pulse pressure played a partial mediating role in the adverse association between TyG index and renal function decline.

**Table 4:** Counterfactual mediation analysis results.

Panel A. Baseline mediation effect estimates					
Effect abbreviation	Estimate	Std Err	P>|z|	95% conf interval lower	95% conf interval upper
cde	-0.8879623	0.11329655	0.000	-1.1100235	-0.665901
nde	-0.8809613	0.11338122	0.000	-1.1031885	-0.65873411
nie	-0.02152459	0.01063233	0.043	-0.04236396	-0.00068521
mte	-0.90248589	0.11339817	0.000	-1.1247463	-0.68022547
Panel B. Bootstrap bias-corrected (BC) mediation effect results					
Effect abbreviation	Observed coefficient	Bias	Bootstrap std. err.	95% BC conf interval lower	95% BC conf interval upper
cde	-0.8879623	-0.0009364	0.13910508	-1.169235	-0.636413
nde	-0.8809613	-0.0012164	0.13909437	-1.163854	-0.6265842
nie	-0.02152459	0.0010806	0.01211679	-0.0558469	-0.0040799
mte	-0.90248589	-0.0001357	0.13954205	-1.181439	-0.6431147

cde, controlled direct effect; nde, natural direct effect; nie, natural indirect effect; mte, marginal total effect; BC, Bias-corrected. Difference between the two panels: Panel A presents the baseline point estimates; asymptotic standard errors; p-values; and 95% asymptotic confidence intervals of the mediation effects; calculated based on the counterfactual mediation linear regression framework. Panel B reports the bias-corrected results based on 1000 bootstrap replications; including the observed coefficient; sampling bias; bootstrap standard error; and 95% bias-corrected confidence interval. This bootstrap approach is applied to correct finite-sample sampling bias and improve the robustness of the estimation results. Covariate adjustment: In addition to the pre-specified covariates in the baseline regression; this model further adjusts for baseline pulse pressure (PP_2011) and baseline estimated glomerular filtration rate (eGFR_2011).

#### 3.3.2 Hospital-based Clinical Validation Cohort (CJFH)

Based on Model 3 (direct effect model) that blocked competing mediated pathways, we further verified the mediating effect of pulse pressure in KDIGO-defined CKD patients. By comparing Model 3 and Model 4 (additional adjustment for baseline pulse pressure), the HR of the TyG index decreased from 1.606 (95% CI [1.196,2.155]) to 1.418 (95% CI [1.030,1.953]), a reduction of 11.69%. Meanwhile, pulse pressure was independently associated with ESRD risk (HR = 1.009, 95% CI [1.001,1.017], P<0.05). This indicated that pulse pressure may play a partial mediating role in the association between TyG index and ESRD in CKD patients, consistent with the exploratory findings of the Community-based Discovery Cohort (**Table 5**).

**Table 5:** Mediating effect of pulse pressure: adjusted cox regression.

Variable	(1)Model 3		(2)Model 4	
	Hazard Ratio	95% confidence intervals	Hazard Ratio	95% confidence intervals
TyG	1.606	[1.196,2.155]	1.418	[1.030,1.953]
PP			1.009	[1.001,1.017]
Gender	1.204	[0.762,1.902]	1.195	[0.758,1.884]
Rural	0.835	[0.539,1.294]	0.846	[0.547,1.308]
Edu	0.824	[0.668,1.016]	0.853	[0.691,1.054]
Marry	0.870	[0.492,1.540]	0.847	[0.480,1.497]
BMI	1.092	[1.058,1.127]	1.089	[1.055,1.124]
Smoke	0.924	[0.590,1.448]	0.945	[0.606,1.474]
Drink	1.075	[0.697,1.656]	1.023	[0.663,1.577]
Stroke	1.728	[0.761,3.926]	1.558	[0.675,3.595]
Hearte	0.793	[0.446,1.410]	0.780	[0.439,1.385]
Diabe	1.067	[0.642,1.771]	1.030	[0.617,1.721]
HDL	0.356	[0.200,0.635]	0.377	[0.213,0.670]
LDL	1.161	[1.020,1.321]	1.144	[1.004,1.304]
Livere	0.440	[0.153,1.266]	0.466	[0.162,1.344]
Kidneye	1.523	[0.788,2.941]	1.663	[0.857,3.229]
Chronic	1.031	[0.666,1.595]	0.987	[0.637,1.529]
Cancer	1.623	[0.641,4.111]	1.664	[0.658,4.209]
Hospital	0.824	[0.492,1.378]	0.852	[0.505,1.436]
Doctor	1.054	[0.943,1.178]	1.055	[0.944,1.179]
Ins	0.951	[0.376,2.404]	0.934	[0.370,2.356]
*N*	396		396	

Results in the CJFH Cohort Exponentiated coefficients (HR) and 95% confidence intervals. Both models are fully adjusted for all covariates. Model 3: Without adjustment for pulse pressure; Model 4: With additional adjustment for baseline pulse pressure. The reduction in the TyG index’s HR (from 1.606 to 1.418) confirms the partial mediating role of pulse pressure.

### 3.4 Moderating effect of hypertension

#### 3.4.1 Community-based Discovery Cohort (CHARLS)

**Figure 5:**
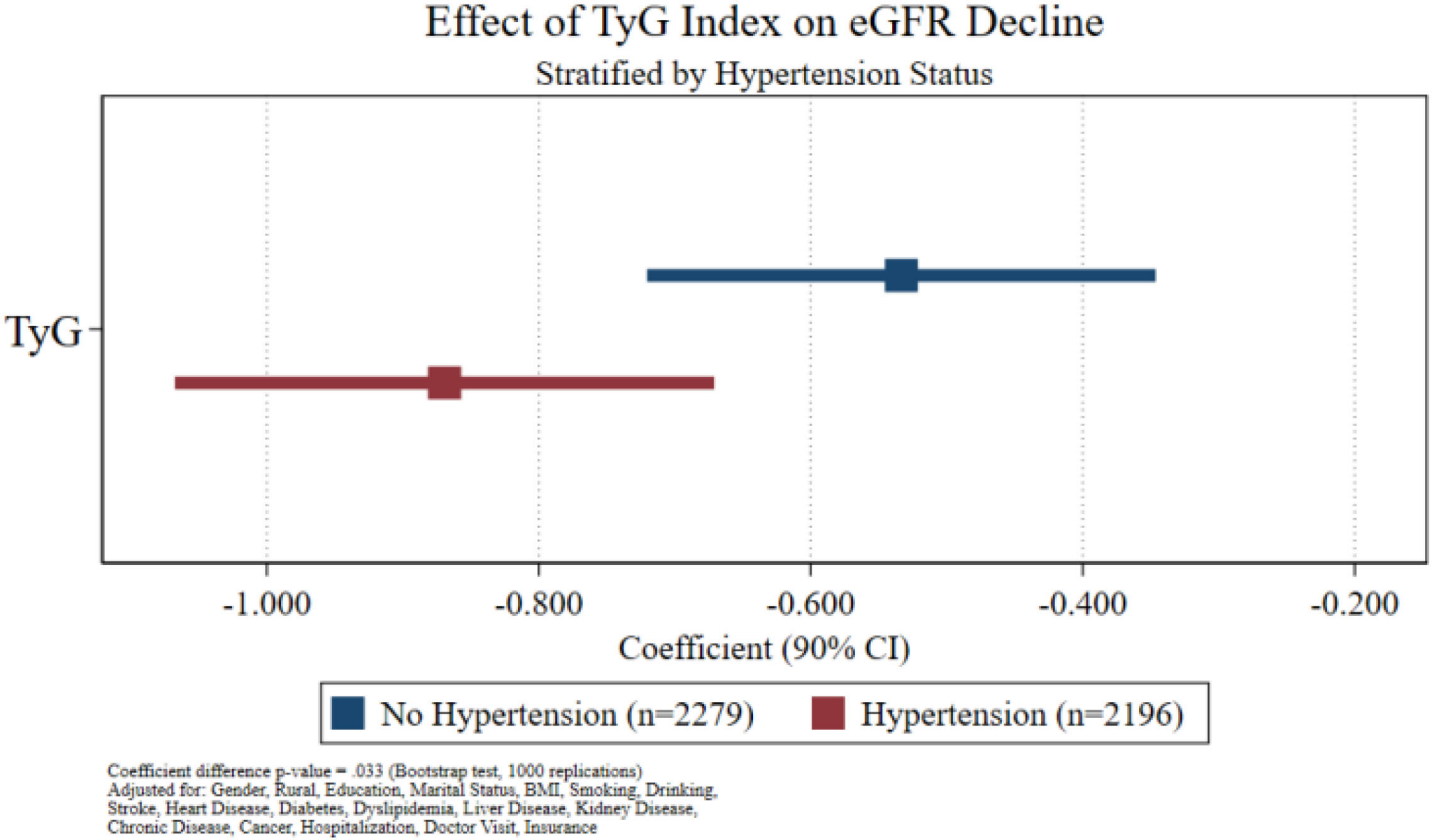
Moderating effect of hypertension. Regression coefficients (90% CI) of the TyG index on eGFR decline stratified by baseline hypertension status in the CHARLS cohort (hypertension group: n=2196; non-hypertension group: n=2279). The TyG coefficient was negative in both groups, with a larger absolute value in the hypertension group. Bootstrap test (1000 repetitions) showed that the difference in coefficients between the two groups was statistically significant (P = 0.033), indicating that hypertension amplifies the adverse effect of the TyG index.

Stratified analysis showed that the absolute value of the regression coefficient of the TyG index on eGFR decline was larger in the hypertension group (n=2196) than in the non-hypertension group (n=2279), and the difference in coefficients between the two groups was statistically significant (P = 0.033), indicating that hypertension amplified the adverse effect of the TyG index (**Figure 5**).

#### 3.4.2 Hospital-based Clinical Validation Cohort (CJFH)

Stratified Cox regression results in KDIGO-defined CKD patients showed that in the non-hypertension group (n=222), the HR of the TyG index on ESRD was 1.405 (95% CI [0.424,4.653], P>0.05), with no significant association. In the hypertension group (n=174), the HR of the TyG index was 1.682 (95% CI [1.198,2.362], P<0.05), meaning that each 1-unit increase in the TyG index was associated with a 68.2% increase in ESRD risk.This confirmed that baseline hypertension significantly amplified the adverse effect of the TyG index on ESRD, consistent with the Community-based Discovery Cohort (**Table 6**).

**Table 6:** Cox regression stratified by baseline hypertension status (CJFH cohort).

Variable	(1)HT=0Group		(2)HT=1Group	
	Hazard Ratio	95% confidence intervals	Hazard Ratio	95% confidence intervals
TyG	1.405	[0.424,4.653]	1.682	[1.198,2.362]
Gender	1.691	[0.803,3.560]	0.884	[0.462,1.693]
Rural	0.833	[0.402,1.725]	0.816	[0.444,1.500]
Edu	0.792	[0.563,1.114]	0.791	[0.594,1.053]
Marry	1.068	[0.453,2.517]	0.658	[0.283,1.531]
BMI	1.111	[1.059,1.165]	1.073	[1.026,1.122]
Smoke	0.979	[0.493,1.947]	0.733	[0.370,1.451]
Drink	1.184	[0.618,2.266]	1.008	[0.520,1.952]
Stroke	5.015	[1.336,18.83]	0.940	[0.281,3.146]
Hearte	1.120	[0.510,2.462]	0.403	[0.148,1.098]
Diabe	1.214	[0.516,2.855]	1.135	[0.553,2.332]
HDL	0.288	[0.109,0.764]	0.342	[0.158,0.739]
LDL	1.285	[1.031,1.600]	1.056	[0.883,1.264]
Livere	0.202	[0.0248,1.642]	0.850	[0.241,2.996]
Kidneye	1.612	[0.569,4.563]	1.711	[0.687,4.257]
Chronic	1.175	[0.615,2.243]	0.655	[0.334,1.285]
Cancer	1.198	[0.336,4.267]	4.318	[0.897,20.79]
Hospital	0.208	[0.0486,0.890]	1.071	[0.642,1.788]
Doctor	1.167	[0.984,1.385]	1.159	[0.886,1.517]
Ins	1.280	[0.297,5.514]	0.609	[0.148,2.516]
*N*	222		174	

HT = 0 Group: Non-hypertension (n=222); HT = 1 Group: Hypertension (n=174). All models are fully adjusted for demographic, lifestyle, disease history, and medical-related factors.

### 3.5 Nonlinear Relationship Between TyG Index and Renal Function Deterioration

#### 3.5.1 Community-based Discovery Cohort (CHARLS)

**Figure 6:**
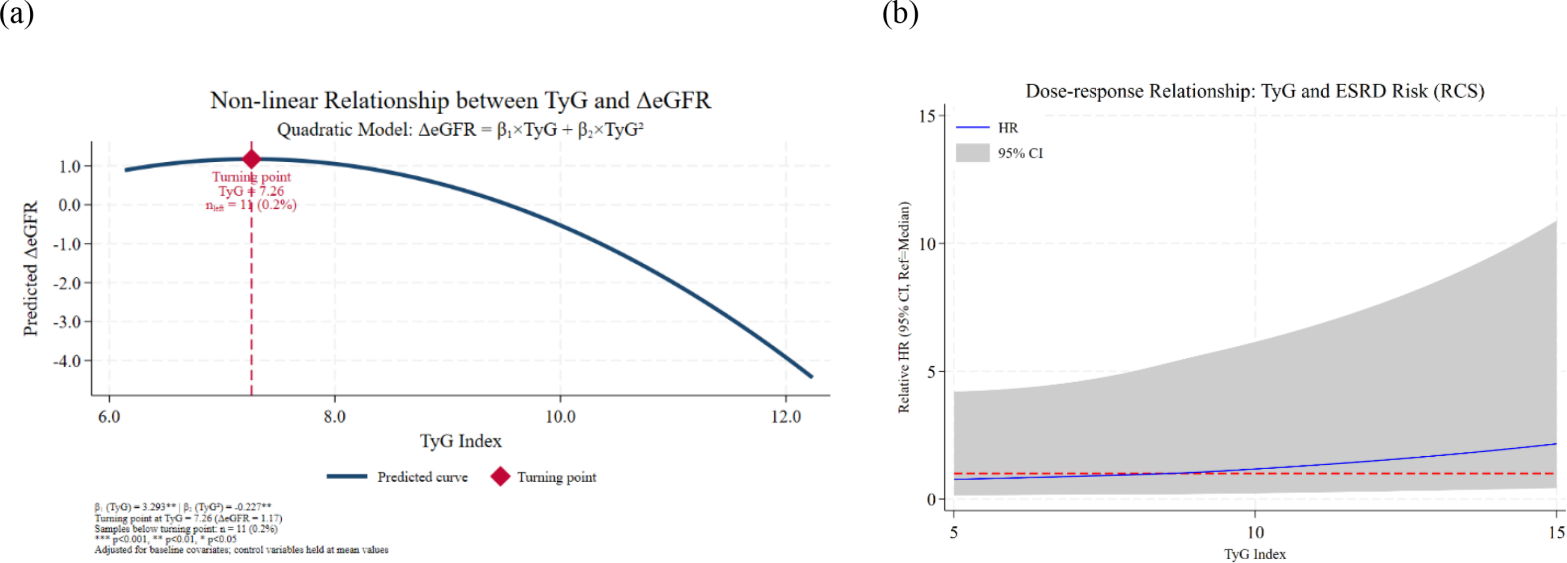
Nonlinear relationship between TyG index and renal function deterioration **(a)** Nonlinear relationship between the TyG index and ΔeGFR in the CHARLS cohort (adjusted for baseline covariates) using a quadratic regression model (ΔeGFR = β_1_×TyG + β_2_×TyG^2^). The linear term coefficient (β₁=3.293, P<0.01) and quadratic term coefficient (β_2_=-0.227, P<0.01) indicated an inverted U-shaped relationship, with an inflection point at TyG=7.26. Only 11 participants (0.2% of the total sample) were below this inflection point, and 99.8% of samples were on the right side, where ΔeGFR continued to decline with increasing TyG, indicating the overall association was dominated by a negative linear trend. **P<0.01. **(b)** Dose-response relationship between the TyG index and ESRD risk in the CJFH cohort using restricted cubic spline (RCS) analysis. The relative hazard ratio (HR) showed a continuous upward trend as the TyG index increased from 5 to 15, with no plateau or downward inflection point. The nonlinear test yielded P = 0.742, confirming no significant nonlinear relationship, and the association was mainly characterized by a linear trend without an obvious “risk threshold” in the TyG range of 5–15. Shaded areas represent 95% confidence intervals.

The multiple linear regression model incorporating the quadratic term of the TyG index showed an inverted U-shaped nonlinear relationship (linear term coefficient=3.293, P<0.01; quadratic term coefficient=-0.227, P<0.01), with an inflection point of 7.26. However, only 11 samples (0.2% of the total) were below this inflection point, and 99.8% of samples were on the right side, where ΔeGFR continued to decline with increasing TyG, indicating that the association was dominated by a negative linear relationship (**Figure 6a**).

#### 3.5.2 Hospital-based Clinical Validation Cohort (CJFH)

Restricted cubic spline analysis showed that as the TyG index increased from 5 to 15, the relative HR showed a continuous upward trend, with no plateau or downward inflection point. The nonlinear test P = 0.7422, indicating no significant nonlinear relationship. Thus, the association between the TyG index and ESRD risk was mainly a linear trend, and there was no obvious “risk threshold” in the range of 5-15. (**Figure 6b**).

Combined with the results of both cohorts, although the Community-based Discovery Cohort showed a statistically significant inverted U-shaped relationship, the inflection point was extremely low and the number of corresponding samples was negligible. Therefore, the overall association between the TyG index and renal function deterioration was dominated by a significant negative linear association.

## 4 Discussion

Based on the Cardiovascular-Kidney-Metabolic (CKM) syndrome framework (8), this study adopted an exploratory-validation two-stage design with two independent prospective cohorts: we explored the long-term predictive value of the TyG index (a validated surrogate for insulin resistance) for early renal function decline in community-dwelling middle-aged and elderly general population via the CHARLS cohort, and validated the adverse effect of TyG index on end-stage renal progression in KDIGO 2012-defined CKD patients via the CJFH cohort. Results indicate that an elevated baseline TyG index is an independent risk factor for renal function deterioration in both cohorts, with pulse pressure (PP) as a potential partial mediator and baseline hypertension as an effect modifier. This study provides epidemiological evidence supporting the core pathophysiology of the “metabolism-vascular-renal” axis of CKM syndrome from both community and clinical settings, while clarifying the heterogeneity of effect size and clinical application scenarios between the two cohorts, and offers stratified insights for early risk stratification of renal impairment in general population and multi-dimensional interventions in CKD patients.

This study established a stable independent association between the TyG index and renal function deterioration. The TyG index was significantly negatively correlated with ΔeGFR in the community-dwelling middle-aged and elderly population from the CHARLS cohort; in the CJFH cohort of KDIGO-defined CKD patients, the TyG index showed a robust adverse effect on ESRD, with each 1-unit increase associated with a 66.2% higher risk in the total effect model (HR = 1.662, 95% CI [1.293,2.138], P<0.001) and a 60.6% higher risk in the direct effect model (HR = 1.606, 95% CI [1.196,2.155], P<0.01); the high TyG tertile (T3) showed a significantly elevated ESRD risk compared to T1 (HR = 1.843, P<0.05) in the direct effect model. This aligns with recent studies (e.g., Shang (7) et al.), but differs in population and association pattern: Shang et al. (7) reported a U-shaped relationship in hospitalized CKM syndrome patients, while our study observed a dominant linear association in community-dwelling middle-aged and elderly population and hospital-based KDIGO-defined CKD patients (nonlinear test P = 0.7422). These differences may stem from population heterogeneity, varying renal injury mechanisms, and distinct comorbidity/therapeutic profiles (7). As the first study exploring the predictive value of TyG index for early renal function decline in Chinese general middle-aged and elderly population and validating the causal association in KDIGO-defined CKD patients, we extend the conclusion that “insulin resistance contributes to both kidney disease occurrence and progression” (9). By using prospective CHARLS data and long-term CJFH follow-up, we clarify the temporal sequence of “insulin resistance exacerbates renal function deterioration,” with robustness confirmed by sensitivity analysis (10). Notably, though an inverted U-shaped nonlinear relationship was detected in the CHARLS cohort (inflection point TyG=7.26), only 0.2% of samples fell below this value—99.8% of included participants showed a linear dose-response between TyG and renal function decline, emphasizing the clinical significance of early TyG-targeted interventions for primary prevention of renal impairment (11).

A key finding of this study is the potential mediating role of pulse pressure in the association between TyG index and renal function deterioration, which provides epidemiological evidence supporting the “metabolism-vascular-renal” cascade hypothesis of CKM syndrome (12). In the CHARLS cohort, we adopted standardized time-anchored SEM and counterfactual framework-based mediation analysis, with all models adjusted for baseline eGFR, baseline pulse pressure and full covariates. The results showed that TyG index may indirectly associate with accelerated eGFR decline in community-dwelling middle-aged and elderly population by increasing pulse pressure, with a statistically significant natural indirect effect (P = 0.043). In the CJFH cohort of KDIGO-defined CKD patients, to isolate the independent mediating effect of pulse pressure, we performed the analysis based on the direct effect model adjusted for competing parallel mediators; adjusting for pulse pressure reduced the HR of TyG index for ESRD by 11.69% (from 1.606 to 1.418), and pulse pressure itself was an independent risk factor for ESRD (HR = 1.009, 95%CI [1.001,1.017], P<0.05), which further supported the exploratory results of the Community-based Discovery Cohort. The underlying mechanism involves three core pathways: insulin resistance activates the sympathetic nervous system and renin-angiotensin-aldosterone system (RAAS) (13) to increase vascular resistance via sodium retention (14); impairs vascular endothelial function by altering nitric oxide bioavailability and endothelin-1 release; and promotes vascular remodeling through chronic inflammation and oxidative stress (15). Widened pulse pressure transmits pulsatile load to the renal microcirculation, causing glomerular hypertension/hyperfiltration (16), structural damage, and intrarenal RAAS activation (15), exacerbating renal injury. This finding suggests that the “TyG-pulse pressure-renal injury” pathway has partial commonality in both general population and CKD patients, but the mediating effect size is different between the two cohorts due to population and disease stage heterogeneity. Interventions targeting insulin resistance may exert renal protection by improving arterial elasticity and reducing pulse pressure in the early stage of renal injury in general population (17); while for CKD patients with established renal parenchymal injury, strategies addressing aortic stiffness and pulse pressure (e.g., RAAS inhibitors, SGLT2 inhibitors) combined with insulin resistance management may be more effective in delaying CKD progression.

This study also confirms baseline hypertension as an important effect modifier: the adverse impact of the TyG index on renal function is significantly amplified in hypertensive KDIGO-defined CKD patients (18). In the CJFH cohort, each 1-unit TyG increase was associated with a 68.2% higher ESRD risk in the hypertensive subgroup (HR = 1.682, P<0.05), but no significant association was observed in non-hypertensive CKD patients (HR = 1.405, P>0.05). This synergy arises from shared pathophysiological pathways (endothelial dysfunction, RAAS activation, inflammation/oxidative stress) (19) that produce a “1 + 1>2” effect, accelerating glomerular injury and renal fibrosis. Clinically, CKD patients with both high TyG and hypertension represent an extremely high-risk group—management should adopt a comprehensive strategy combining blood pressure control (prioritizing RAAS inhibitors with metabolic benefits) and insulin resistance improvement (lifestyle interventions, insulin sensitizers), aligning with the holistic CKM syndrome management approach (18).

Notably, there are inherent biological and clinical heterogeneities between the CHARLS and CJFH cohorts, which should be clearly clarified to avoid over-simplified integration and over-interpretation of consistent mechanisms. First, in terms of population characteristics, the CHARLS cohort is community-dwelling middle-aged and elderly general population, focusing on the early stage of renal function impairment in the natural population; while the CJFH cohort is hospital-based KDIGO-defined CKD patients with baseline eGFR <60 mL/min/1.73 m², focusing on the progression of established renal disease to end-stage renal failure. The biological basis of renal damage is significantly different: the former is mostly early hemodynamic and metabolic injury without definite structural damage, while the latter has established renal parenchymal injury and fibrosis, with more complex comorbidities and compensatory mechanisms. Second, in terms of study design, the CHARLS cohort has a fixed 4-year follow-up, with the outcome of continuous change in eGFR (ΔeGFR) to reflect early renal function decline; the CJFH cohort has a median follow-up of 1019 days, with the hard clinical endpoint of ESRD, which reflects the terminal outcome of renal function deterioration. Third, in terms of statistical design, the covariate adjustment strategy of the two cohorts is different: the CHARLS cohort focuses on controlling demographic, lifestyle and basic disease confounding factors in the general population, while the CJFH cohort further classifies covariates into pre-exposure confounders and parallel mediators according to the causal pathway of CKD progression, to more accurately isolate the effect of TyG index in the clinical population with established renal disease. Based on the above heterogeneities, this study does not suggest that the mechanism of TyG index affecting renal function is completely consistent between the two cohorts; instead, the two cohorts complement each other to reveal the continuous effect of insulin resistance on renal function from the early pre-disease stage in the general population to the end-stage progression in CKD patients, with partial commonality in the core pathophysiological pathway of “metabolism-vascular-renal” axis.

Despite rigorous design, this study has limitations. First, as an observational study, it cannot fully rule out unmeasured confounders or reverse causality, even with prospective data design, strict inclusion and exclusion criteria, multi-factor adjustment for common demographic, lifestyle and disease-related confounders, and multiple sensitivity analyses (including double machine learning model) to verify the robustness of the results. We have excluded or adjusted for most common confounding factors that may affect the study results, but the inherent limitations of observational studies cannot be completely eliminated. Second, for the CHARLS cohort, urinary albumin data were not fully available, and CKD status was not confirmed by repeated eGFR measurements as required by the KDIGO guidelines, which may lead to potential classification bias; thus, the results of this cohort are only used for exploratory analysis of early renal function decline prediction in the general population, and cannot be extrapolated to confirmed CKD patients. Third, eGFR (estimated via CKD-EPI formula) and the TyG index are surrogate indicators, introducing potential measurement errors (20). Fourth, generalizability is limited to middle-aged/elderly Chinese community-dwelling population and hospitalized KDIGO-defined CKD patients—extrapolation to younger populations or other ethnic groups requires caution. Fifth, mediating effect analysis relies on statistical assumptions (21), and real-world biological pathways may be more complex (e.g., bidirectional interactions between pulse pressure and insulin resistance, unmeasured mediators) (22). Future studies should use more precise measurements and randomized controlled trials to validate these associations and evaluate the clinical value of multi-organ protective drugs (e.g., SGLT2 inhibitors, GLP-1 receptor agonists) in this high-risk population.

## 5 Conclusion

This study adopts an exploratory-validation two-stage design with two independent longitudinal observational cohorts, providing epidemiological evidence supporting the CKM syndrome theoretical framework across the full spectrum from general population to confirmed CKD patients: the community-based CHARLS cohort reveals that insulin resistance (characterized by the TyG index) is closely associated with long-term early renal function decline in middle-aged and elderly general population; the hospital-based CJFH cohort of KDIGO-defined CKD patients validates that elevated TyG index is significantly associated with ESRD progression. The adverse association between TyG index and renal function deterioration may be partially mediated by increased pulse pressure and synergistically amplified by baseline hypertension, with partial commonality in the core pathophysiological pathway of “metabolism-vascular-renal” axis, rather than completely consistent action mechanisms between the two cohorts due to inherent population and disease stage heterogeneities. These findings suggest that clinical management should adopt a stratified strategy based on population characteristics: for the general middle-aged and elderly population, TyG index can be used as a routine screening indicator for early risk stratification of renal function impairment; for CKD patients confirmed by KDIGO guidelines, management should go beyond a single-organ perspective and integrate the assessment of metabolic and vascular health. Early identification and intervention of insulin resistance, strict control of pulse pressure, especially targeting the high-risk phenotype of “high TyG index combined with hypertension”, may be a core strategy for delaying renal function deterioration. In the future, prospective studies using more precise measurement indicators and repeated eGFR confirmation in line with KDIGO guidelines are needed to further validate the population-specific associations; at the same time, randomized controlled trials targeting CKM syndrome should be designed to evaluate the clinical value of multi-organ protective drugs such as SGLT2 inhibitors and GLP-1 receptor agonists in different populations.

## Data Availability

The original contributions presented in the study are included in the article/supplementary material. Further inquiries can be directed to the corresponding author.

## Appendix: Sensitivity Analysis of the Community-based Discovery Cohort (CHARLS)

To verify the robustness of the core conclusions of the Community-based Discovery Cohort, multiple sensitivity analyses were designed for “measurement bias, residual confounding, and sample selection bias”:

1. **Replacement of the measurement method of the dependent variable**: The percentage change in eGFR (ΔeGFR%=(eGFR in 2015 - eGFR in 2011)/eGFR in 2011×100%) and ΔeGFR calculated using the CKD-EPI formula based on serum cystatin C (SCys-C) were used respectively. SCys-C is less affected by muscle mass and diet, which can reduce the measurement error in renal function assessment.
2. **Inclusion of urban fixed effects**: To control for macro-level confounding factors such as differences in medical resource allocation and public health policies among different cities, improving the external validity of the results nationwide.
3. **Restriction of the sample range**: Only patients with ΔeGFR <0 were retained to focus on the target population with “confirmed renal function decline” and exclude the diluting effect of individuals with no change on effect estimation.
4. **Double machine learning model**: Integrating 6 algorithms including neural network, random forest, gradient boosting, Lasso regression, ridge regression, and elastic network to simultaneously capture the linear/nonlinear effects and interactions of covariates. This addresses the strict assumption limitations of traditional regression models on the distribution of covariates, further verifying the robustness of the TyG index effect.
